# *Ex Vivo COL7A1* Correction for Recessive Dystrophic Epidermolysis Bullosa Using CRISPR/Cas9 and Homology-Directed Repair

**DOI:** 10.1016/j.omtn.2018.06.008

**Published:** 2018-06-26

**Authors:** Araksya Izmiryan, Clarisse Ganier, Matteo Bovolenta, Alain Schmitt, Fulvio Mavilio, Alain Hovnanian

**Affiliations:** 1 Laboratory of Genetic Skin Diseases, INSERM UMR 1163, Imagine Institute, 24 bd du Montparnasse, Paris, France; 2 University Paris Descartes-Sorbonne Cité, Paris, France; 3 INSERM UMR 951, Genethon, Evry, France; 4 Electronic Microscopy Facility, INSERM UMR 1016, Cochin Institute, Paris, France; 5 Department of Life Sciences, University of Modena and Reggio Emilia, Modena, Italy; 6 Imagine Institute, Paris, France; 7 Department of Genetics, Necker Hospital for Sick Children, APHP, Paris, France

**Keywords:** recessive dystrophic epidermolysis bullosa, *COL7A1*, CRISPR/Cas9, gene targeting, homology-directed repair, primary keratinocytes and fibroblasts, droplet digital PCR, skin grafts

## Abstract

Recessive dystrophic epidermolysis bullosa is a rare and severe genetic skin disease resulting in blistering of the skin and mucosa. Recessive dystrophic epidermolysis bullosa (RDEB) is caused by a wide variety of mutations in *COL7A1*-encoding type VII collagen, which is essential for dermal-epidermal adhesion. Here we demonstrate the feasibility of *ex vivo COL7A1* editing in primary RDEB cells and in grafted 3D skin equivalents through CRISPR/Cas9-mediated homology-directed repair. We designed five guide RNAs to correct a RDEB causative null mutation in exon 2 (c.189delG; p.Leu64Trpfs*40). Among the site-specific guide RNAs tested, one showed significant cleavage activity in primary RDEB keratinocytes and in fibroblasts when delivered as integration-deficient lentivirus. Genetic correction was detected in transduced keratinocytes and fibroblasts by allele-specific highly sensitive TaqMan-droplet digital PCR (ddPCR), resulting in 11% and 15.7% of corrected *COL7A1* mRNA expression, respectively, without antibiotic selection. Grafting of genetically corrected 3D skin equivalents onto nude mice showed up to 26% re-expression and normal localization of type VII collagen as well as anchoring fibril formation at the dermal-epidermal junction. Our study provides evidence that precise genome editing in primary RDEB cells is a relevant strategy to genetically correct *COL7A1* mutations for the development of future *ex vivo* clinical applications.

## Introduction

Dystrophic epidermolysis bullosa (DEB) is a rare and severe genetic skin disease of children and adults, responsible for blistering of the skin and mucosa. DEB is caused by a wide variety of mutations in the *COL7A1* gene-encoding type VII collagen.^1^ Type VII collagen is the major component of anchoring fibrils (AFs), which are key attachment structures for dermal-epidermal adhesion.^2^ *COL7A1* mutations lead to defective AF formation, resulting in a loss of adhesion between the epidermis and the dermis.^3^ Depending on the nature and position of *COL7A1* mutations, the disease is either dominantly or recessively inherited,^4^ dominant forms (DDEB) being usually less severe than recessive forms. Generalized severe recessive DEB (RDEB) is one of the most severe genodermatoses in children.^5^ Individuals with RDEB undergo continuous wound healing and tissue repair, often associated with significant local and systemic complications, leading to mutilating scarring, contracture deformities, fusion of fingers and toes, chronic skin inflammation, anemia, malnutrition, and a dramatically increased risk of developing squamous cell carcinomas at sites exposed to continuous wounding.^6^ No specific cure is currently available for RDEB and proposed treatments are mainly symptomatic.^7^

Recent gene-targeting studies have highlighted the potential of precise correction of disease-causing mutations in primary cells by site-specific nucleases, such as meganucleases (MNs), zinc-finger nucleases (ZFNs), transcription activator-like effector nucleases (TALENs), or CRISPR/Cas9.^8^, ^9^, ^10^, ^11^ These nucleases can induce DNA double-strand breaks (DSBs) at specific loci and stimulate homology-directed repair (HDR) in the presence of a Donor template.^12^, ^13^, ^14^

During the last 5 years, we and others could achieve gene editing in RDEB cells using nuclease-based approaches.^15^, ^16^, ^17^, ^18^ First, Osborn and colleagues^15^ obtained up to 2% of genetic correction in RDEB patient-derived fibroblasts through TALEN-mediated HDR. Using antibiotic selection, corrected cells were reprogrammed into induced pluripotent stem cells (iPSCs), and type VII collagen rescue was demonstrated by *in vivo* teratoma assay. We reported up to 4% of *COL7A1* correction of two disease-causing mutations using MN-mediated HDR in an RDEB patient-derived keratinocyte cell line and in primary RDEB keratinocytes (RDEB-Ks) and fibroblasts (RDEB-Fs).^16^ Recently, Hainzl and colleagues achieved genetic and functional correction of a disease-causing mutation in an RDEB-K cell line through CRISPR/Cas9-mediated HDR using antibiotic- or fluorescence-based selection. Rescue of C7 was demonstrated in skin grafts composed of genetically corrected keratinocytes’ cell line without evidence of AF formation at the dermal-epidermal junction.^18^

The achievements described above represent important steps in gene correction for RDEB through nuclease-mediated HDR. However, several major drawbacks prevent their direct translation to the clinic. These include the low efficiency of gene correction using sequence-specific nucleases such as MNs or TALENS, the limited efficacy of non-viral delivery methods to skin cells, and the use of antibiotic- or fluorescence-based selection to enrich targeted cell populations. These limitations need to be overcome in order to consider the use of genetically corrected transplantable skin grafts for future clinical applications.

In this study, we developed an *ex vivo* gene therapy approach based on CRISPR/Cas9-mediated *COL7A1* editing without antibiotic- or fluorescence-based selection. We achieved efficient *COL7A1* editing in primary RDEB-Ks and RDEB-Fs, and we demonstrate functional rescue of type VII collagen expression and AF formation in an *ex vivo* xenograft model using gene-edited RDEB skin grafts.

## Results

### Design of Guide RNAs Targeting Exon 2 of *COL7A1*

**Figure 1:**
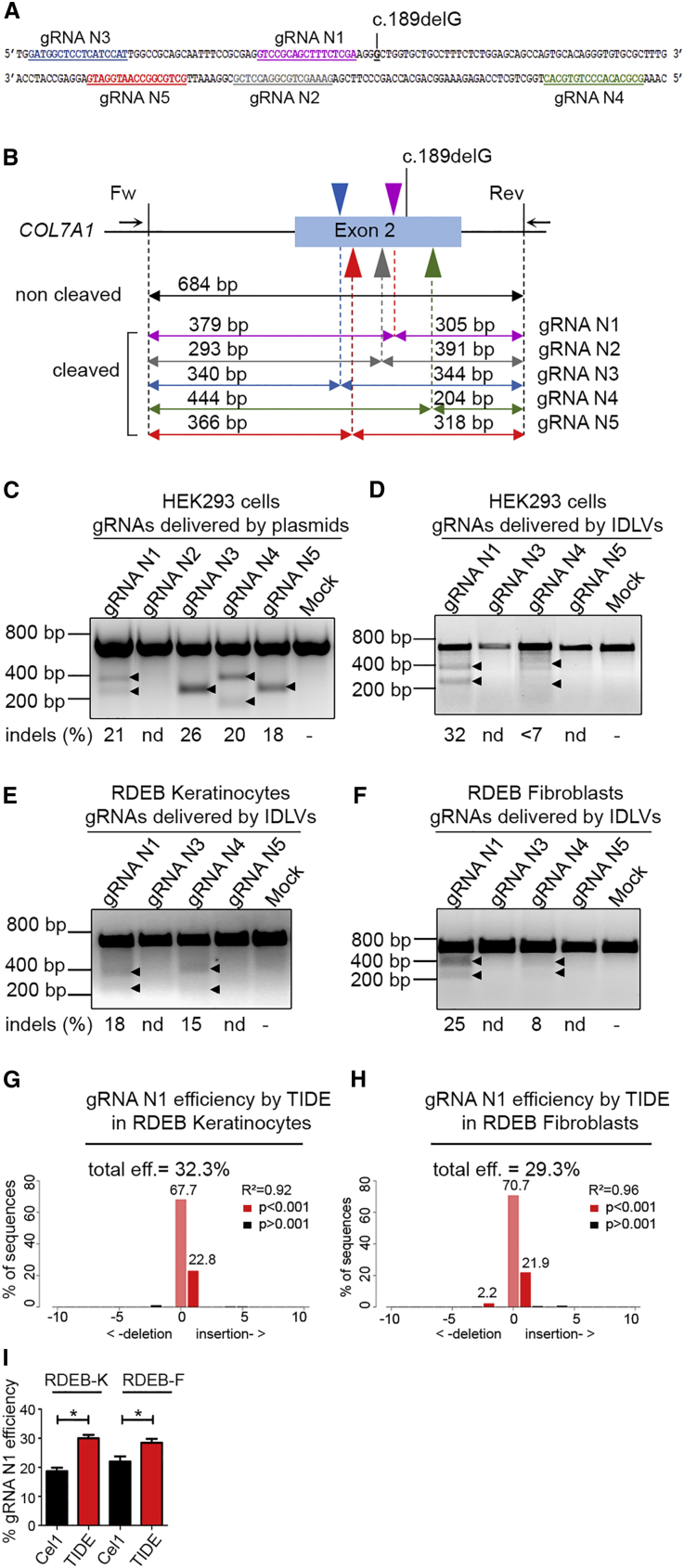
Design and Evaluation of the Cleavage Activity of gRNAs Targeting *COL7A1* Exon 2 (A) Sequences of the human *COL7A1* exon 2 showing the location of five different gRNAs (N1–N5; colored and underlined in violet, gray, blue, green, and red) upstream or downstream of the c.189delG mutation. (B) PCR strategy for the Surveyor cleavage assay. Top: target sites of gRNAs (arrowheads) located in exon 2 are shown. Arrows indicate forward and reverse PCR primers generating the 684-bp amplicon. Bottom: surveyor cleavage theoretically produces the following bands: 305 and 379 bp for gRNA N1, 293 and 391 bp for gRNA N2, 340 and 344 bp for gRNA N3, 240 and 444 bp for gRNA N4, and 318 and 366 bp for gRNA N5. (C) Evaluation of the activity of gRNAs specific for exon 2 delivered together with Cas9 nuclease by plasmids. HEK293T cells were transfected with both plasmids, one encoding for each of the gRNAs and the second encoding for the Cas9 nuclease. 72 hr later, gDNA was extracted and analyzed with the Surveyor cleavage assay. Up to 22% of NHEJ activity was scored for gRNAs except gRNA N2. (D–F) Evaluation of the activity of gRNAs delivered by IDLVs. HEK293T cells (D), primary RDEB keratinocytes (RDEB-Ks) (E), and fibroblasts (RDEB-Fs) (F) were treated with IDLVs containing Cas9 together with gRNA (N1, N3, N4, or N5) and similarly analyzed 72 hr later. Arrowheads indicate the cleaved bands. Only gRNAs N1 and N4 showed NHEJ activity when delivered by IDLVs. The percentage of modification by NHEJ (indels) indicated below each lane was determined by densitometry. nd, non-detected. (G and H) Evaluation of the cleavage activity of gRNA N1 encoding IDLVs by Sanger sequencing followed by TIDE analysis in RDEB keratinocytes (RDEB-Ks) (G) and fibroblasts (RDEB-Fs) (H). (I) Comparison of the cleavage activity of gRNA N1 estimated by the Surveyor (Cel I) and TIDE assay with p values according to Student’s t test. Values are represented as mean ± SD; *p = 0.0243 for RDEB-Ks and *p = 0.03 for RDEB-Fs. Each experiment was repeated 3 times.

To demonstrate the feasibility of *COL7A1* editing in primary RDEB cells using the CRISPR/Cas9 approach, we designed five guide RNAs (gRNAs) in order to correct a causative null mutation in exon 2 seen in several RDEB subjects (c.189delG; p.Leu64Trpfs*40). To avoid or to limit off-target events, we used gRNAs with improved design (17- to 18-bp length instead of 20 nt).^19^ Five gRNAs were identified by ZiFiT Targeter 4.2 (http://zifit.partners.org) software in the human genome (Figure 1A). Target sites of these gRNAs are located in close distance (∼5–44 bp downstream or upstream) from the mutation to be corrected (Table S1). No other sequence within the human genome showed 100% identity to these target sites.

### Activity Assessment of gRNAs Targeting Exon 2 of *COL7A1*

First, the selected five gRNAs (gRNA N1 to gRNA N5) were cloned into an expression plasmid and delivered together with a plasmid encoding for Cas9 in HEK293T cells to assess their activity and specificity.

The capacity of gRNAs to induce site-specific DNA cleavage was assessed by documenting non-homologous end joining (NHEJ) at the targeted region. Indeed, gRNAs can induce targeted DNA DSBs, which can be repaired by the NHEJ pathway in the absence of a recombination template. This results in the introduction of small insertions or deletions (indels) at the target locus, which can be scored by the Surveyor mismatch-sensitive cleavage assay (Figure 1B). To evaluate the activity of gRNAs, HEK293T cells were transfected with similar doses of gRNA-encoding plasmids together with Cas9 nuclease. NHEJ was monitored 72 hr after transfection. Genomic DNA was extracted, and the targeted region was amplified by PCR and subjected to the Surveyor nuclease (Figure 1C).

Among the five gRNAs tested, four showed up to 26% activity in HEK293T cells. Only gRNA N2 did not show activity in HEK293T cells. These four active gRNAs were then cloned into an HIV-derived lentiviral vector (lentiCRISPR-v2) and delivered as integration-deficient lentiviruses (IDLVs) in order to efficiently transduce primary RDEB-Ks and RDEB-Fs. Similarly, HEK293T cells, RDEB-Ks, and RDEB-Fs were transduced with IDLVs encoding for gRNA N1, N3, N4, or N5 at the dose of 1.5 pg p24/cell, and gene modification frequency was monitored at day 3 after transduction. Significant differences in NHEJ rates were observed. Among the four site-specific gRNAs tested, only gRNAs N1 and N4 showed activity in HEK293T cells (32% and 7%), in primary RDEB-Ks (18% and 15%), and in RDEB-Fs (15% and 8%) when delivered as IDLVs, respectively (Figures 1D–1F). We observed no activities of gRNAs N3 and N5 delivered as IDLVs, which could be explained by (1) the possibility that the gRNA in the context of an IDLV genome in the pre-integration complex of lentiviral vectors has a limited stability in contrast to transfected naked plasmid DNA in cells, and (2) inefficient IDLV packaging.

In addition, we assessed NHEJ induced by the gRNA N1 encoding IDLVs by Sanger sequencing and Tracking of Indels by Decomposition (TIDE) analysis, and we found similar targeting efficiencies estimated to be 32.3% and 29.3% in RDEB-Ks and RDEB-Fs, respectively (Figures 1G and 1H). Discrepancies between values of the NHEJ frequency evaluated by the Surveyor cleavage assay and the TIDE assay may be explained by the underestimation of NHEJ activity when using the Surveyor assay, which is less sensitive than the TIDE assay.^20^ Overall, gRNA N1 showed the highest activity in transduced cells, and it was, therefore, considered for further gene correction experiments.

### Genetic Correction of *COL7A1* c.189delG Mutation by CRISPR-Mediated HDR in Primary RDEB-Ks and RDEB-Fs

**Figure 2:**
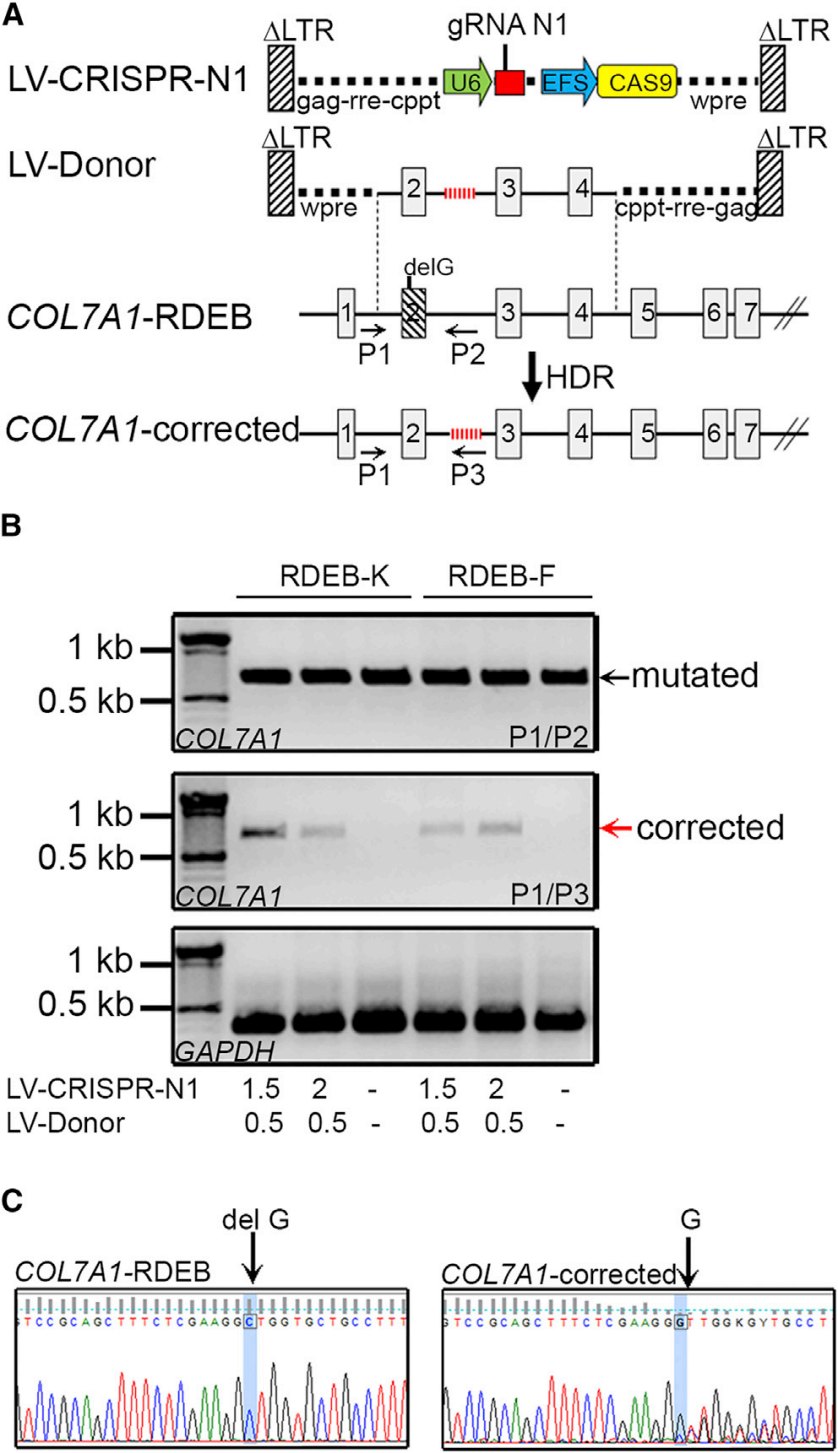
CRISPR/Cas9-Mediated HDR at the *COL7A1* Locus (A) Top: design of the lentiviral vectors (LV-CRISPR-N1 encoding for gRNA N1 together with Cas9 and LV-Donor encoding for a promoterless Donor construct containing homology sequences spanning the regions between introns 2 and 4). In this construct, the 6-bp tag inserted in intron 2 allows allele-specific amplification after HDR). Middle: *COL7A1* genomic organization encompassing mutated exon 2. Bottom: structure of corrected *COL7A1* after CRISPR/Cas9-mediated HDR. Arrows indicate the location of primers for corrected allele-specific genomic PCR. (B) Genomic PCR analysis of the edited *COL7A1* locus. Primary RDEB-Ks and RDEB-Fs were transduced with the indicated doses of virus (pg p24 per cell), and the gDNA was extracted at 21 days post-transduction and analyzed by PCR. A specific 0.8-kb band corresponding to the corrected allele was amplified when using P1/P3 primers. No specific amplification was detected in mock (untreated) cells, as expected. The GAPDH gene was used as a reference. (C) Sequencing of the PCR product generated with the P1/P3 primers from gene-edited RDEB-Ks revealed genetic correction of the c.189delG mutation through CRISPR/Cas9-mediated HDR.

To achieve efficient correction of the c.189delG (p.Leu64Trpfs*40) null mutation in exon 2, primary RDEB-Ks and RDEB-Fs, from a patient homozygous for this mutation, were treated with increasing doses of IDLVs encoding gRNA N1 together with the Donor template and analyzed at 21 days post-transduction (Figure 2A). This late time point was considered to avoid episomal expression of IDLVs encoding the Donor template during the first days of treatment, which is gradually lost by dilution in dividing cells after transduction.^21^ Genomic DNA was extracted and gene correction was investigated on bulk-transduced cells using two strategies: allele-specific genomic PCR and droplet digital TaqMan PCR. The first strategy detects only HDR events because the PCR primers are specific for the genomic sequence (at their 5′ end) and the modified Donor sequence (at their 3′ end) beyond the HDR junction. Gene correction occurred only in primary RDEB-Ks and RDEB-Fs treated with both LV-CRISPR-N1- and LV-Donor-encoding IDLVs (LV-CRISPR-N1, 1.5 or 2 pg p24/cell; and LV-Donor, 0.5 pg p24/cell). In contrast, untreated cells did not show the presence of the corrected band (Figure 2B). Direct sequencing of the PCR product from RDEB-Ks generated with the P1/P3 primer set confirmed restoration of the normal genomic sequence (Figure 2C).

**Figure 3:**
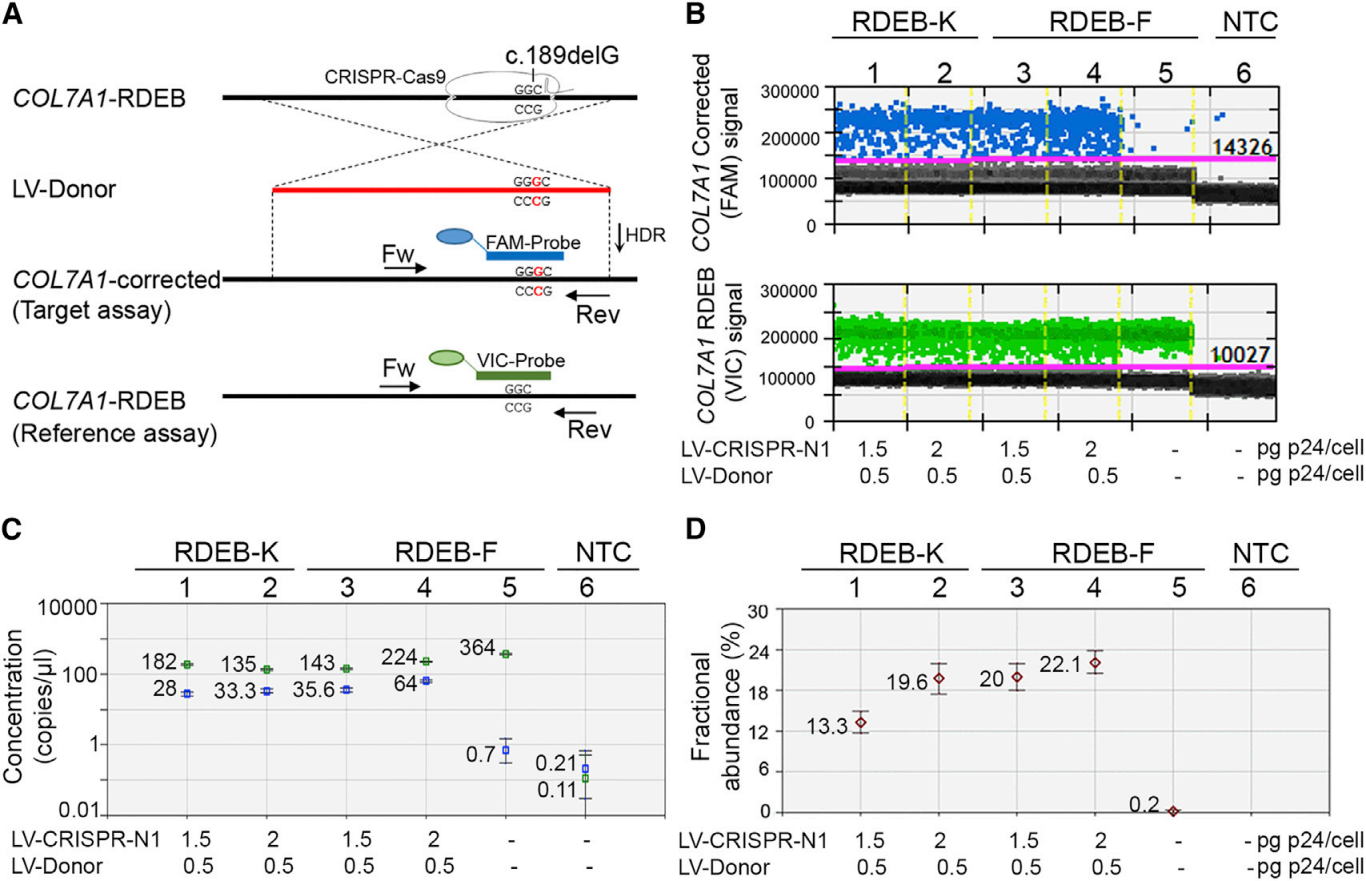
TaqMan-ddPCR-Based Detection of CRISPR/Cas9-Mediated HDR at the *COL7A1* Locus (A) Gene editing detection strategy for *COL7A1* by ddPCR on the gDNA level. The location of a common primer pair and allele-specific probes conjugated with FAM (specific for the corrected *COL7A1*) or VIC (specific for the mutated *COL7A1*) fluorophores are indicated. (B) Primary RDEB-Ks and RDEB-Fs were transduced with the indicated doses of IDLVs (pg p24 per cell). 21 days post-transduction, gDNA was extracted, amplified, and analyzed by ddPCR to assess allelic frequency of corrected *COL7A1* on the mutated background. 1D fluorescence amplitude plots are shown for FAM and VIC signals. Blue dots correspond to the FAM signal amplitude and represent droplets containing the corrected *COL7A1* alleles. Green dots correspond to the VIC signal amplitude and represent the mutated *COL7A1* alleles. Gray dots correspond to empty droplets. Yellow lines indicate borders between different samples. (C and D) Quantification of positive droplets using Quantasoft Software. The concentration plot (C), showing gene-edited wells of FAM and VIC amplicons, is automatically determined by the software using the total number of events (displayed in Figure S1C) by correcting for Poisson distribution. The blue markers indicate corrected *COL7A1* copies/μL and the green markers indicate mutated *COL7A1* copies/μL. The fractional abundance plot (D) shows the percentage of frequency of the corrected *COL7A1* on the mutated *COL7A1* background. All error bars were generated by QuantaSoft and represent a 95% confidence interval.

The second strategy combines a TaqMan assay with the recently developed ultra-sensitive droplet digital PCR (ddPCR) technology, and it allows performing an absolute quantification of *COL7A1* editing after CRISPR/Cas9-mediated HDR at the gDNA level. The detection sensitivity was estimated to be as low as 0.02%.^22^ For this assay, we designed a common primer pair and allele-specific TaqMan probes conjugated with FAM and VIC fluorophores that distinguish between the corrected (target assay) and the mutated sequences (reference assay), respectively (Figure 3A; Figure S1A). To screen for *COL7A1* editing, 50 ng genomic DNA from RDEB-Ks and RDEB-Fs, transduced with increasing doses of virus (LV-CRISPR-N1, 1.5 or 2 pg p24/cell; and LV-Donor, 0.5 pg p24/cell), was subjected to TaqMan-ddPCR. The assay was performed in duplex conditions (FAM and VIC probes were added in the same reaction per sample), and the allelic frequency of the corrected *COL7A1* (FAM+) and the mutated *COL7A1* (VIC+) was investigated. Gene-edited alleles (FAM+) were easily detected and quantified by QuantaSoft software (Figures 3B–3D; Figures S1B and S1C). Transduced RDEB-Ks showed 13.3% and 19.6% of editing (wells N1 and N2), and transduced RDEB-Fs showed 20% and 22% of editing (wells N3 and N4), depending on the doses of IDLVs. A very small number of positive droplets, found for the sample with the DNA from untreated cells (well N5) or the sample without DNA template (well N6), was considered to be false positives (Figure 3D).

Further, we performed TaqMan-ddPCR to evaluate the efficiency of *COL7A1* correction at the mRNA level (Figures S2A–S2D). For this assay, we used two probes: the target probe (FAM labeled), which recognizes the corrected *COL7A1* (the same as described above, Figure 3), and the reference probe (VIC labeled), which recognizes the housekeeping *RPLP0* gene. The percentage of the corrected *COL7A1* transcript in RDEB-Ks (well N1) and RDEB-Fs (well N5) was calculated to be 11% and 15.7%, respectively (Figure S2D). In contrast, cells treated only with the Donor (wells N2 and N6) did not show significantly positive amplification, while untreated cells (wells N3 and N7) were completely negative (Figures S2B–S2D).

### Rescue of Type VII Collagen Expression in Gene-Edited RDEB-Ks and RDEB-Fs

**Figure 4:**
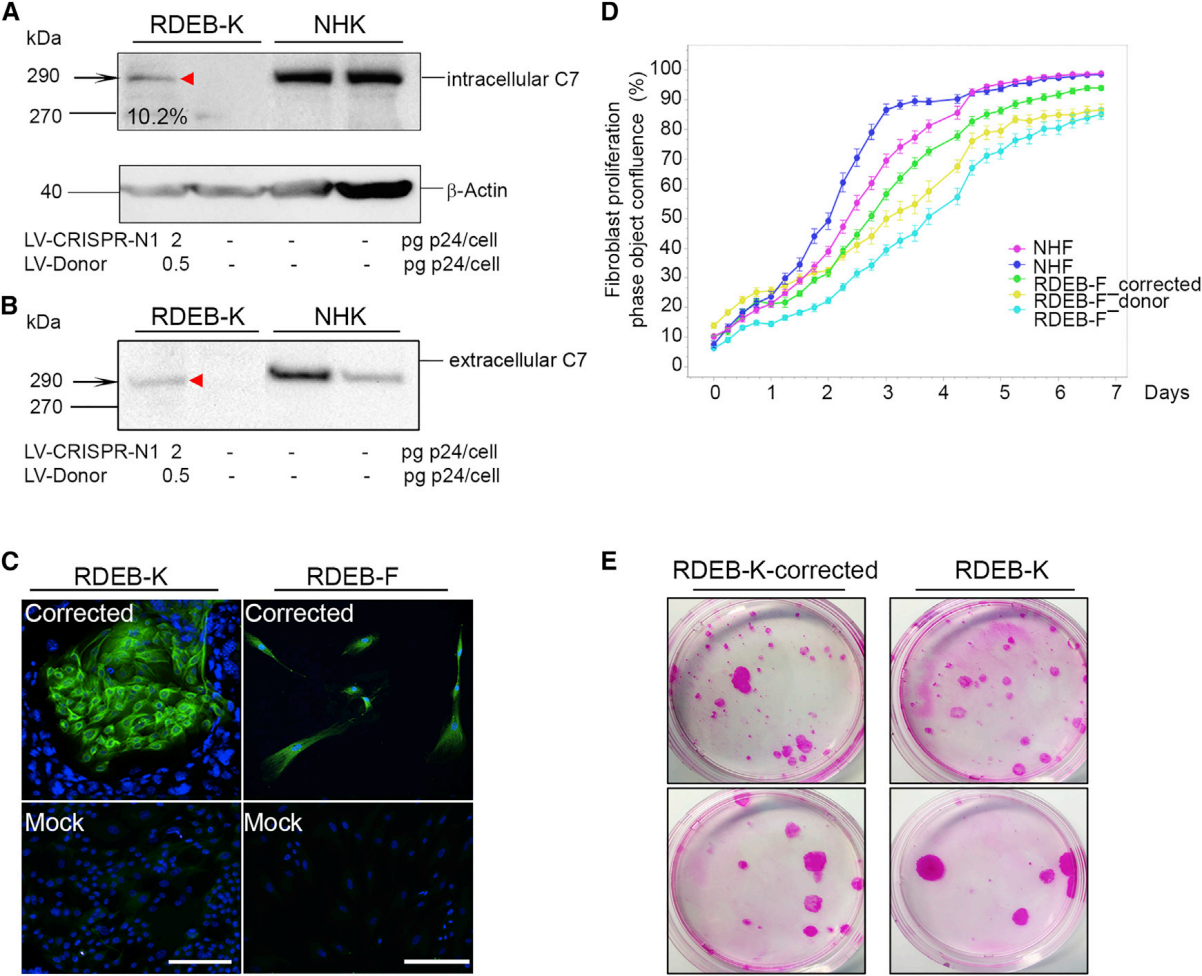
Restoration of Type VII Collagen Expression in Gene-Edited RDEB Keratinocytes and Fibroblasts (A) Western blot analysis showing C7 re-expression in lysates from gene-edited RDEB-Ks using the LH7.2 anti-C7 antibody. Antibody to β-actin served as the loading control. (B) Western blot analysis showing secreted C7 in conditioned medium from gene-edited RDEB-Ks using the LH7.2 anti-C7 antibody. Normal human keratinocytes (NHKs) show a normal level of C7 expression in lysates and in conditioned medium. (C) Gene-edited RDEB-Ks and RDEB-Fs 21 days post-transduction, positively stained for type VII collagen. Untreated (mock) cells show negative staining for C7 as expected. Scale bars, 50 μm. (D) Gene-edited fibroblast growth and confluence monitoring using IncuCyte. NHFs, untreated primary RDEB-Fs, and RDEB-Fs treated with CRISPR-N1 together with or without the Donor-encoding IDLVs were seeded in 12-well plates and monitored during 8 days. Data were analyzed using IncuCyte S3 basic analyzer software. NHF confluence monitoring is presented in dark blue or pink. Corrected RDEB-Fs (in green) show selective advantage of proliferation in comparison with untreated RDEB-Fs (in light blue) or RDEB-Fs treated only with Donor-encoding IDLVs (in yellow). (E) Colony-forming efficiency assay on gene-corrected RDEB keratinocytes and untreated RDEB keratinocytes 21 days post-transduction at a density of 500 (upper panel) or 100 cells (lower panel) in 10-cm Petri dishes coated with irradiated 3T3-J2 MEF as a feeder layer.

To investigate restoration of type VII collagen expression after CRISPR/Cas9-mediated HDR, we performed western blotting and immunocytochemistry analysis on transduced primary RDEB-Ks using anti-type VII collagen LH7.2 antibody (Figure 4). First, we assessed C7 protein expression and secretion in gene-corrected cells versus normal human keratinocytes (NHKs) treated with increasing doses of IDLVs encoding for the LV-CRISPR-N1 and the Donor template. Intracellular C7 protein expression was detected from cell extracts (Figure 4A) and was estimated to be 10.66% and 11% (compared to C7 expression in NHKs), depending on the doses of IDLVs (LV-CRISPR-N1, 1.5 or 2 pg p24/cell; and LV-Donor, 0.5 pg p24/cell) (Figure 4A). In parallel, C7 protein secretion was detected from conditioned media (Figure 4B), when high doses of virus were used (LV-CRISPR-N1, 2 pg p24 per cell). In contrast, untreated RDEB cells were negative for C7. Rescued C7 expression was also confirmed by immunocytochemistry on primary RDEB-Ks and fibroblasts, co-treated with the IDLVs encoding for LV-CRISPR-N1 and the Donor template, whereas untreated RDEB cells (mock) completely lacked C7 (Figure 4C). Thus, *COL7A1* editing and desired phenotypic correction (up to 11%) could be achieved in primary RDEB-Ks and RDEB-Fs by HDR through combined gRNA- and Donor-encoding IDLV delivery.

In parallel, to evaluate whether non-integrative lentiviral vector-based gene correction modifies the morphology and the proliferative capacity of transduced primary RDEB-Fs, we monitored gene-corrected fibroblast growth and confluence using the IncuCyte live-cell-imaging system. Corrected RDEB-Fs showed a selective advantage of proliferation in comparison with untreated RDEB-Fs or RDEB-Fs treated only with Donor-encoding IDLVs (Figure 4D). No difference in morphology was found between normal human fibroblasts (NHFs) and treated or untreated RDEB-Fs (data not shown).

We have also investigated the regenerative capacity of gene-corrected RDEB-Ks by performing a colony-forming efficiency assay. We found that the lentiviral vector-based transduction did not impair the keratinocyte proliferative capacity in comparison with untreated RDEB-Ks (Figure 4E).

### Analysis of Off-Target Sites and the Persistence of Cas9 in Gene-Edited RDEB Cells

**Figure 5:**
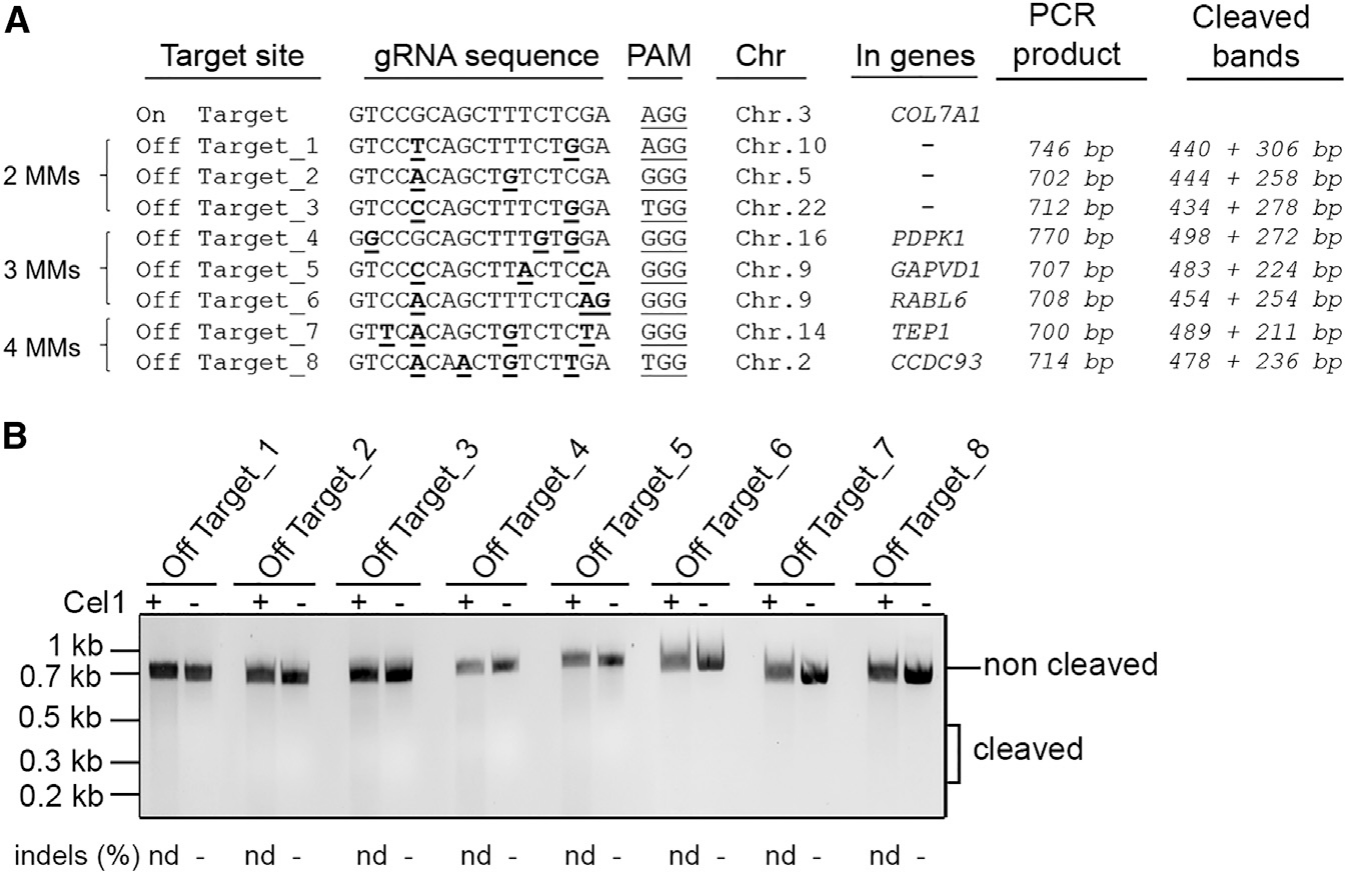
Off-Target Site Analysis (A) Potential off-target cleavage hits for the gRNA N1 were identified by ZiFiT Targeter 4.2 in the human genome. Mismatches (MMs) are underlined. Note that there is no off-target with 1 MM. 3 potential off-targets were found with 2 MMs in non-coding regions. In addition, 3 potential off-targets with 3 MMs and 2 potential off-targets with 4 MMs were identified in coding regions. The 8 off-target sequences were subjected to the Surveyor cleavage assay. (B) Genomic DNA from RDEB-Ks treated with IDLVs encoding for the LV-CRISPR-N1 (2 pg p24 per cell) was extracted, and regions corresponding to off-target sequences were amplified by PCR using specific primers (listed in Table S4). The Surveyor cleavage assay was performed at each potential off-target hit. No Surveyor activity indicative of cleavage at predicted off-target sites was detected. nd, non-detected.

To investigate whether gRNA N1 triggers potential off-targets in edited RDEB cells, we looked for human genomic sequences displaying significant homology with the cognate site. Potential off-target sequences for gRNA N1 were identified by ZiFiT Targeter 4.2 (http://zifit.partners.org) in the human genome, and only 3 potential off-targets (Off-Targets_1,2,3) were found with 2 mismatches (MMs) in non-coding regions. Off-target prediction using http://crispr.mit.edu identified 3 additional potential off-targets with 3 MMs (Off-Targets_4,5,6) and 2 potential off-targets with 4 MMs (Off-Targets_7,8) in coding regions (Figure 5A). Thus, to determine if the *COL7A1*-specific gRNA N1 was able to induce DSBs at non-specific loci bearing partial homology with the endogenous target sequence listed above, RDEB cells were treated with the gRNA N1-encoding IDLVs (1.5 pg p24 per cell), genomic DNA was extracted at 2 days post-transduction, and regions corresponding to these 8 off-target sequences were amplified by PCR and digested by the Surveyor nuclease. Results showed no evidence for non-specific cleavage (∼350-bp expected band after digestion) of selected off-target hits at sites with partial homology (Figure 5B). Moreover, no evidence for non-specific cleavage of the same off-target hits was found in genetically corrected RDEB-Ks 21 days post-transduction (Figure S3).

To investigate the persistence of Cas9 in genetically corrected cells after transduction, we performed absolute quantification assay, and we found very few copies of Cas9 cDNA in these cells. Up to 1.8 Cas9 copies/μL were found in bulk-transduced cells, corresponding to 2.25 × 10^−6^ and 3.81 × 10^−6^ Cas9 copies/cell in RDEB-Ks and RDEB-Fs, respectively. To investigate the persistence of Cas9 in genetically corrected cells after transduction, we performed absolute quantification assay, and we found very few copies of Cas9 cDNA (up to 1.8 copies/μL) in these cells. This may be explained by the fact that episomal or integrated forms of IDLVs could be still present in these cells 21 days post-transduction. Importantly, we found no evidence for the persistence of residual Cas9 cDNA in genetically corrected skin equivalents (SEs) 1 and 2 months post-grafting (Table S2).

### *Ex Vivo* Functional Correction of Type VII Collagen and AF Formation at the Dermal-Epidermal Junction

To assess functionality of type VII collagen in gene-corrected RDEB-Ks and RDEB-Fs, we produced 3D SEs and analyzed them in a human: murine xenograft model. Three groups of SEs were produced and grafted onto the dorsal region of immunodeficient mice using a skin flap procedure as previously reported.^23^, ^24^ The first group was composed of primary NHKs and NHFs, the second was composed of corrected primary RDEB-Ks and RDEB-Fs, and the third was composed of untreated primary RDEB-Ks and RDEB-Fs. At 4 and 10 weeks later, skin grafts were removed and subjected to immunofluorescence, molecular, and ultrastructural analysis.

**Figure 6:**
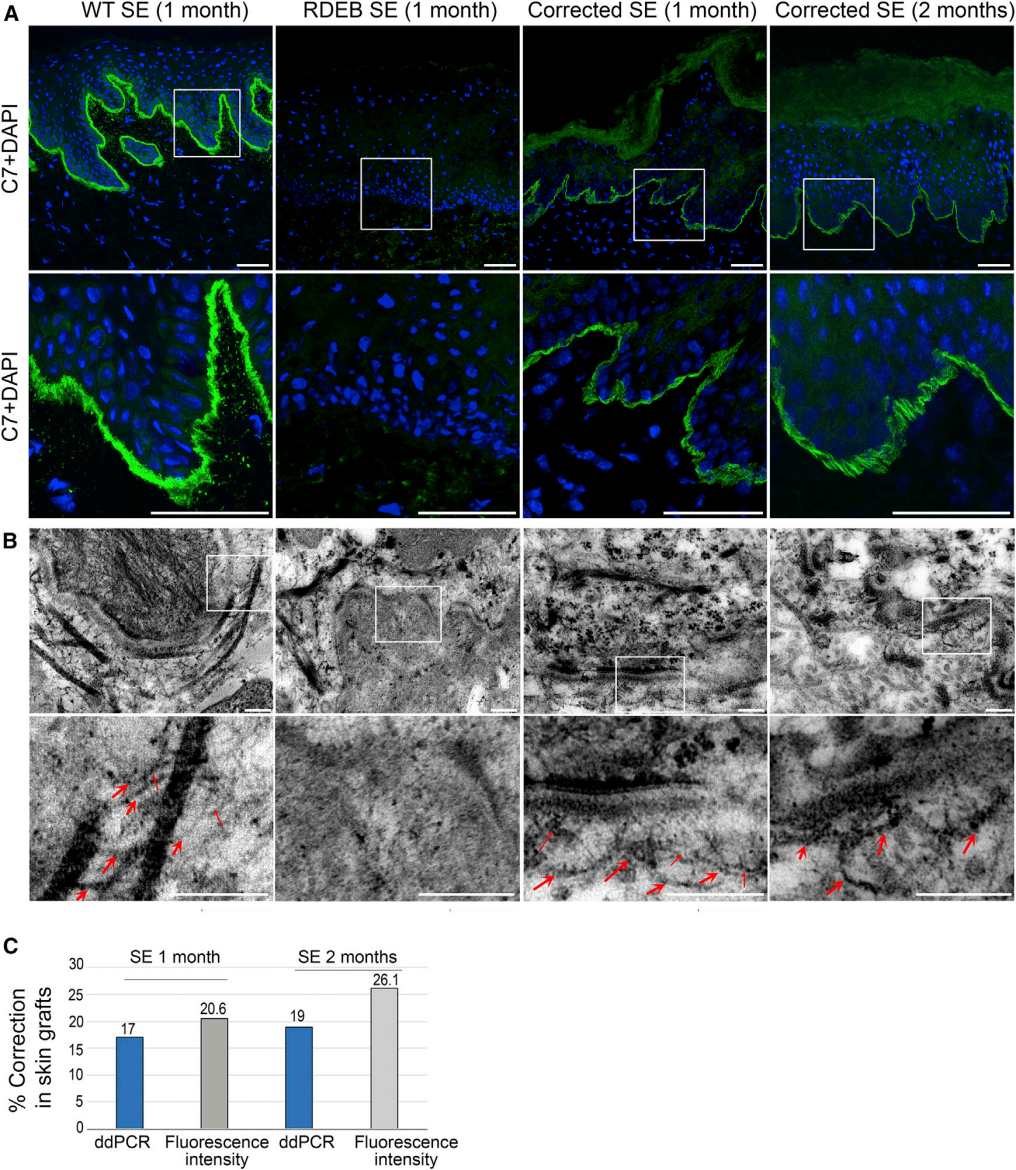
Type VII Collagen Rescue, Localization, and AF Formation at the Dermal-Epidermal Junction in Genetically Corrected Skin Grafts (A) Immunofluorescence analysis of grafted SE composed of normal, RDEB, or genetically corrected primary RDEB keratinocytes and fibroblasts at 1 or 2 months after deflapping. Skin samples composed of genetically corrected cells showed re-expression and normal localization of C7 at the dermal-epidermal junction in contrast to skin samples composed of untreated RDEB keratinocytes and fibroblasts, totally devoid of C7. Scale bars, 50 μm. (B) Ultrastructural analysis of skin grafts by transmission electron microscopy (TEM). Anchoring fibrils showing thick, cross-binding structural characteristics inserted into lamina densa were found in skin samples composed of genetically corrected cells at 1 or 2 months after deflapping. Anchoring fibrils are indicated by red arrows. Scale bars, 200 nm. (C) Percentage of C7 correction in skin grafts quantified by ddPCR (blue bars, see also Figure S3) and by mean intensity of fluorescence using ImageJ software (gray bars).

Immunofluorescence analysis on the serial sections of skin grafts composed of genetically corrected primary RDEB-Ks and RDEB-Fs showed C7 rescue and linear localization at the dermal-epidermal junction similar to the normal skin (Figure 6A; Figure S5). In contrast, skin grafts made from untreated RDEB-Ks and RDEB-Fs were totally devoid of C7. Up to 26% of C7 rescue was found at the dermal-epidermal junction in skin grafts by quantifying mean fluorescence intensity of type VII collagen stained with LH7.2 antibody (Figure 6C, gray bars). At the genomic DNA level, up to 19% of *COL7A1* editing was found on cryosections of skin grafts by ddPCR (Figure 6C, blue bars; Figure S6). A very small number of positive droplets found in the sample of skin grafts composed of untreated cells or in the sample without DNA template (non-treated control [NTC]) was considered to be false positive (Figure S6). Finally, analysis of ultrathin sections of genetically corrected skin grafts by transmission electron microscopy revealed the presence of structures characteristic of AFs at the dermal-epidermal junction (Figure 6B). They showed funning and central cross-banding structures forming loops around dermal collagen fibers and inserted into the lamina densa, similar to AFs seen in healthy skin grafts composed of NHKs and NHFs. In contrast, RDEB skin grafts composed of untreated cells showed no evidence for AFs (Figure 6B).

## Discussion

Recently developed gene-, cell-, protein-, or bone marrow transplantation- based strategies for DEB have shown therapeutic potential with variable efficacy and safety.^23^, ^24^, ^25^, ^26^, ^27^, ^28^, ^29^ The first successful gene therapy report for junctional EB (JEB) caused by *LAMB3* mutations used autologous grafts of epithelial sheets genetically corrected with a classical γ-retroviral vector expressing *LAMB3* cDNA.^30^ Recently, the same vector was used to genetically correct keratinocytes of a 7-year-old child suffering from JEB. He was almost entirely (80%) grafted with autologous genetically corrected epidermal sheets and showed fully functional epidermis.^31^ Specifically, gene addition approaches for RDEB currently involve self-inactivating (SIN) lentivirus- or retrovirus-mediated transfer of *COL7A1* cDNA into keratinocytes and/or fibroblasts and transplantation of corrected epithelia or SEs with restored type VII collagen protein expression, leading to AF formation and recovering of dermal-epidermal adherence.^24^, ^27^, ^32^ A phase 1 *ex vivo* gene therapy clinical trial for RDEB using transplantation of autologous epithelial sheets genetically modified with a classical retroviral vector expressing *COL7A1* cDNA was conducted at Stanford.^27^ Among four patients treated, three showed improved wound healing of grafted sites and type VII collagen expression localizing to AFs at the dermal-epidermal basement membrane at least 1 year post-treatment, without significant adverse events.

In several pre-clinical studies, the therapeutic potential of intradermally injected gene-corrected RDEB-Fs was also investigated. Intradermal injections of RDEB-Fs genetically corrected with a therapeutic-grade SIN lentiviral vector, encoding codon-optimized *COL7A1*, showed functional restoration and AF formation at the dermal-epidermal junction in human RDEB skin xenografts on NOD-scid IL2Rgamma-null mice.^29^ Our group showed that intradermal injections of genetically corrected RDEB-Fs using a SIN retroviral vector also showed functional restoration and AF formation at the dermal-epidermal junction in a xenograft RDEB SE model in nude mice.^23^ Both vectors have been proposed for clinical trials using local intradermal injections of autologous gene-corrected fibroblasts. Although the use of SIN vectors considerably reduces the risks of cell transformation by oncogene activation, aberrant splicing, epigenetic modifications, or progressive extinction of the therapeutic transgene may still occur.^33^, ^34^

In this context, gene correction approaches based on sequence-specific DSB-mediated HDR offer a major advantage in the field of gene therapy as compared to conventional strategies.^35^, ^36^ They allow precise correction of disease-causing mutations restoring physiologic expression and function of the gene by preserving the endogenous transcriptional regulation.^37^ Recently, gene editing proof-of-concept approaches were successfully achieved in RDEB^15^, ^16^, ^17^ as well as in HIV infection, alpha-1 antitrypsin (AAT) deficiency, cystic fibrosis, Duchene muscular dystrophy, X-linked severe combined immunodeficiency syndrome, hemophilia B, sickle cell anemia, and Fanconi anemia.^38^, ^39^, ^40^, ^41^, ^42^, ^43^, ^44^ Finally, nuclease-mediated HDR has been shown to be more effective in iPSCs than in primary skin cells, which could open new perspectives for the treatment of RDEB and various other genetic diseases.^45^, ^46^ Such genetically corrected cells could then be used to generate 3D SEs to demonstrate functional and sustained correction through type VII collagen protein expression and AF formation.^47^, ^48^, ^49^ The relevance of iPSCs combined with gene correction approaches has been demonstrated in two RDEB murine models,^50^, ^51^ in naturally occurring revertant human RDEB-Ks,^52^ and in fibroblasts from DDEB patients.^53^

In our study, we provide evidence for efficient functional *COL7A1* repair by using patient’s primary keratinocytes and fibroblasts. The homozygous c.189delG (p.Leu64Trpfs*40) null mutation in exon 2 selected for this study was identified in several RDEB patients with severe generalized RDEB (unpublished data). We used IDLVs to deliver the specific gRNA together with Cas9 nuclease and the Donor template in RDEB cells as these vectors have been previously reported as being effective to achieve homologous recombination in human epithelial stem cells.^54^ The exon2-specific gRNA that we designed led, in the presence of the repair template, to the genetic correction of the *COL7A1* mutation and to functional restoration of type VII collagen expression. Analyses at the cDNA and protein levels showed evidence for precise editing at the mutated locus with 11% and 15.7% of corrected *COL7A1* transcripts in transduced primary keratinocytes and fibroblasts, respectively. In our study, the Surveyor (Cel 1) cleavage assay indicated the absence of a detectable level (threshold >1%–5%) of off-target gene modification. In fact, to avoid or to limit these events, we used gRNAs with improved design (high specificity score and short size; 17- to 18-bp length instead of 20 nt).^19^ However, genome-wide analysis of off-targeting is necessary before clinical translation, as non-specific targeting can cause genome instability and disruption of a normal gene function.^55^, ^56^, ^57^ The use of high-fidelity or SIN Cas9 nucleases (spCas9-HF1, eSpCas9_1.1, HypaCas9, KamiCas9),^57^, ^58^, ^59^, ^60^ Cas9 nickase instead of nuclease,^61^, ^62^ or recently described Adenine base editors (ABEs convert A*T to G*C) could be considered to reduce off-target breaks.^63^

To our knowledge, only low efficiency of gene correction (2%–4%) could be achieved through MN- or TALEN-mediated HDR in primary RDEB cells or immortalized keratinocyte cell lines without selection.^15^, ^16^ Recently, *COL7A1* editing was also obtained through CRISPR/Cas9-mediated HDR in a RDEB-K cell line using antibiotic- and fluorescence-based selection. Type VII collagen rescue was demonstrated in skin grafts composed of genetically corrected immortalized keratinocytes, but there was no evidence of AF formation at the dermal-epidermal junction.^18^ It is believed that ≥35% of normal C7 levels are necessary for skin mechanical stability based on wild-type (WT) fibroblast injection experiments in a DEB hypomorphic murine model.^28^ In our study, we could demonstrate that re-expression of *COL7A1* transcripts estimated to be up to 11% and 15% in RDEB cells and up to 19% in skin grafts was sufficient to allow AF formation with no dermal-epidermal separation.

Most likely explanations include the following: (1) *in vivo* positive selection of C7-expressing epidermal clones (Figure 4C), (2) local diffusion of C7 at the sites of synthesis (Figure 6A; Figure S5), and (3) possible contribution of gene-corrected fibroblasts with increased capacities of proliferation (Figure 4D). Of note, gene-edited keratinocytes prior to transplantation showed 11% of *COL7A1* correction at the genomic DNA level, as shown by ddPCR (Figure S2D). These cells expressed up to 11% of C7 on western blot (Figures 4A and 4B). Following transplantation, immunofluorescence of gene-corrected SEs showed 20%–26% of C7 expression in a continuous linear staining pattern along the dermal-epidermal junction (Figure 6A). In addition, droplet PCR of genomic DNA extracted from skin sections of grafted gene-corrected RDEB SEs showed up to 19% of *COL7A1* correction (Table S3). These results are in favor of local diffusion of C7 at the sites of synthesis and its accumulation at the dermal-epidermal junction.

Moreover, we did not find evidence for the persistence of residual Cas9 expression in genetically corrected SEs 1 and 2 months post-grafting (Table S2).

Overall, our data provide evidence for efficient and functional *ex vivo* correction of *COL7A1* in a human: murine xenograft model. Therefore, precise genome editing for RDEB is a new and promising strategy to correct *COL7A1* recurrent mutations and sequences located nearby, for the development of future *ex vivo* clinical applications.

## Materials and Methods

### gRNA Design and Plasmid Construction

gRNAs were designed using the ZiFiT Targeter 4.2 (http://zifit.partners.org) online software. Five gRNA (N1–N5) sequences targeting *COL7A1* (Table S1) were cloned into the plasmid MLM3636 (Addgene 43860) according to the available guidelines. The activity of gRNAs was tested using the plasmid PX165 encoding for the human codon-optimized Cas9 nuclease from Streptococcus pyogenes (SpCas9) (Addgene 48137). Selected gRNAs displaying activity in HEK293T cells were cloned into the plasmid lentiCRISPR_v2 (Addgene 52961) according to the available guidelines to efficiently transduce RDEB-Ks and RDEB-Fs. The LV-Donor lentiviral vector, used for gene-targeting experiments, was described previously.^16^

### Lentiviral Vector Production and Titration

gRNAs and the Donor template were delivered as IDLVs, pseudotyped with the vesicular stomatitis virus G protein (VSV-G) envelope glycoprotein. Lentivirus production was performed as described previously.^64^ Briefly, HEK293T cells were transfected with the relevant lentiviral construct (LV-CRISPR-N1, LV-CRISPR-N3, LV-CRISPR-N4, LV-CRISPR-N5, or LV-Donor) and packaging plasmids pBA-Rev, p-Tat, p-MDG, or p-HDMHg/pM2-D64L (for IDLV production) by calcium phosphate precipitation. Culture media were harvested after 48 and 72 hr, passed through a 0.45 μm filter (Millipore), and ultracentrifuged at 100,000 × *g* for 2 hr. Pellets were re-suspended in PBS-BSA 1% and stored at −80°C. For lentivirus titration, p24Gag was measured using the QuickTiter lentivirus titer kit (Euromedex). Lentivirus titer was calculated according the general guideline of 1 ng p24 = 10^5^ transducing units (TUs). Yield ranged from 1 to 10 μg/mL p24 corresponding to 10^8^–10^9^ TUs/mL.

### Assessment of CRISPR/Cas9 Activity by Surveyor Cleavage Assay and TIDE Analysis

The capacity of gRNAs to induce DSBs at its target site was evaluated by Surveyor cleavage assay (Integrated DNA Technologies), according to the manufacturer’s instructions. Briefly, HEK293T cells and primary RDEB-Ks and RDEB-Fs were transduced with 1.5 pg (p24/cell) gRNA-Cas9-encoding IDLVs. After 72 hr, genomic DNA was extracted using Genomic DNA from tissue kit (Macherey-Nagel), and a 0.7-kb region flanking the gRNA target site was amplified by PCR (Phusion high-fidelity DNA polymerase, New England Biolabs) using specific primers (see Table S4). The following conditions for amplification were used: 98°C for 2 min; then 29 cycles at 98°C for 30 s, 62°C for 30 s, and 72°C for 45 s; followed by extension at 72°C for 5 min. Untreated cells were used as controls. 200 ng purified PCR products were denatured, digested with the nucleotide-mismatch-sensitive Cel1 endonuclease, and analyzed on 1.5% agarose gel using ImageLab (Bio-Rad) software. The intensities of the bands were quantified by ImageJ. The percentage of NHEJ was calculated using the formula 100 × (1 − (1 − fraction cleaved)^1/2^), where the fraction cleaved is the ratio of the intensity of the cleaved bands (digested bands) over the cleaved and un-cleaved bands for each experimental setting.

In parallel, PCR products amplified from RDEB-Ks and RDEB-Fs treated with IDLVs encoding for the gRNA N1 were sequenced by Sanger and analyzed by the TIDE web tool (https://tide.nki.nl), as initially described by Brinkman and colleagues.^65^

### Cells

HEK293T cells and primary RDEB-Fs were maintained in DMEM with Glutamax and 4.5 g/L glucose (Life Technologies) supplemented with 10% fetal bovine serum (FBS) (Lonza) and 1% antibiotics (Life Technologies). Primary RDEB-Ks were maintained in a keratinocyte growth medium, a DMEM-Glutamax and Ham’s F12 (Life Technologies) media mixture (3:1) containing 10% FBS, 1% antibiotics, adenine (0.18 mM), insulin (5 μg/mL), hydrocortisone (0.4 μg/mL), cholera toxin (0.1 nM), triiodothyronine (0.2 nM), and epidermal growth factor (EGF, 10 ng/mL). Primary RDEB-Ks were plated on lethally irradiated mouse 3T3-J2 fibroblasts and grown in keratinocyte growth medium (before and after transduction) or cultivated in Epilife medium (after transduction) with 0.06 mM calcium and human keratinocyte growth supplement (HKGS) containing a final concentration of 0.2% bovine pituitary extract (BPE), 5 μg/mL bovine trasferrin, 0.18 μg/mL hydrocortisone, 5 μg/mL bovine insulin, and 0.2 ng/mL EGF (Life Technologies).

### HDR Assay

Primary RDEB-Ks and RDEB-Fs were seeded at 10^5^ cells/well in 12-well plates. The following day (or 2 days later only for primary keratinocytes grown on irradiated 3T3J2 feeder layer), cells were co-infected with the indicated doses of vectors in the presence of 8 μg/mL polybrene (Sigma-Aldrich). After 72 hr, cells were harvested, reseeded in 6-well plates, and grown for at least 21 days.

### Analysis of Genetically Corrected Cells by Allele-Specific PCR

Genomic DNA from transduced cells was isolated and 200 ng was used for PCR to detect corrected or mutant allele after HDR. To investigate the correction of exon 2 in primary RDEB-Ks and RDEB-Fs, a common P1 forward primer in combination with P2 or P3 specific reverse primers was used to detect mutant or corrected allele, respectively. The GAPDH gene was used as a reference (Table S4). The following conditions for amplification were used: 98°C for 2 min; then 34 cycles at 98°C for 30 s, 56°C for 30 s, and 72°C for 30 s; followed by extension at 72°C for 5 min. PCR products were analyzed on 1% agarose gel and imaged with ImageLab (Bio-Rad). Corrected allele-specific PCR product from gene-corrected keratinocytes was subjected to Sanger sequencing.

### Analysis of Genetically Corrected Cells by TaqMan-ddPCR

For the TaqMan-ddPCR assay, sequences of designed primers and probes specific for corrected and mutated *COL7A1* are shown in Table S4. The ddPCR assay was performed following the manufacturer’s instructions (Bio-Rad). Each ddPCR reaction consisted of a 20-μL solution containing 10-μL ddPCR supermix for probes (Bio-Rad), 900 nM primer, 250 nM FAM-labeled probe, 250 nM VIC-labeled probe, and 50 ng template DNA. About 20,000 droplets were generated per reaction on the Bio-Rad QX-100 Droplet Generator. Then the emulsion was transferred into a 96-well PCR plate for standard PCR on a T100 Thermal Cycler (Bio-Rad). The PCR program was as follows: initial denaturation at 95°C for 10 min, followed by 40 cycles of melting at 95°C for 30 s and annealing /extension at 60°C for 1 min (Rampe Rate at 2°C/s), and final enzyme deactivation at 98°C for 10 min. After cycling, the 96-well plate was immediately transferred into the Bio-Rad QX-100 Droplet Reader, where flow cytometric analysis determined the fraction of PCR-positive droplets (blue and green dots) versus the number of PCR-negative (gray dots) droplets in the sample. Data were analyzed by QuantaSoft Software (Bio-Rad) using the RED (rare events detection) option. The concentration of target versus reference DNA was automatically determined and corrected for Poisson distribution. All error bars were generated by the software and represent 95% confidence interval.

To evaluate the efficiency of *COL7A1* editing in transduced RDEB cells after CRISPR/Cas9-mediated HDR, 50 ng genomic DNA of corresponding samples was subjected to ddPCR. The assay was performed using primers and probes complementary to the corrected or the mutated sequence in exon 2 of *COL7A1* (custom design). The allelic frequency of corrected versus mutated *COL7A1* was determined in the fractional abundance plot, which shows the percentage frequency of the corrected *COL7A1* on the mutated *COL7A1* background.

To evaluate the level of corrected *COL7A1* mRNA expression, total RNA was extracted from transduced primary RDEB cells using RNeasy Mini Kit (QIAGEN), and cDNA was synthesized using SuperScript IV First-Stand Synthesis System (Thermo Scientific). Gene expression assay was performed using primers and probe complementary to the corrected sequence in exon 2 of *COL7A1* (as described above) and the *RPLP0* (reference 99999902; Thermo Scientific), the latter amplification used as normalizer. *COL7A1* mRNA was normalized to the housekeeping gene control *RPLP0*. No specific amplification in RDEB-Ks and RDEB-Fs was found. The relative expression level of the corrected *COL7A1* mRNA was calculated on the basis of the ratio plot (concentration of *COL7A1* versus *RPLP0*) by considering it in normal cells (NHKs and NHFs) as 100%.

### Western Blot Analysis

For intracellular detection of C7, keratinocytes grown in the presence of 20 ng/mL transforming growth factor β2 (Abcam, Cambridge, UK) were lysed in a buffer, containing 150 mmol/L NaCl, 50 mmol/L Tris-HCl (pH 8), 5 mmol/L EDTA, 1% NP40, and complete mini protease inhibitor cocktail (Roche, Basel, Switzerland), 72 hr after transduction. Supernatants were precipitated by the addition of 3 vol cold acetone and incubated at −20°C for 48 hr. The following day, precipitated proteins were pelleted by centrifugation and solubilized in a Tris-Urea (25-mmol/L, 9.5-mol/L) buffer. 60 mg proteins in a 4× Laemmli buffer was loaded, denatured at 70°C for 10 min, separated on 3%–8% precast SDS-PAGE gels (Life Technologies), and transferred to nitrocellulose membrane. The LH7.2 antibody was used (1:1,000) to detect type VII collagen. β-actin immunodetection served as a loading control. Signals were revealed with ECL-plus reagent (Thermo Scientific) using ImageLab software (Bio-Rad).

For the detection of secreted C7, keratinocytes were grown for 48 hr in the presence of 20 ng/mL transforming growth factor β2 and 50 μg/mL ascorbic acid in a serum-free keratinocyte growth medium. The medium was collected, precipitated, and analyzed using LH7.2 antibody as described above.

### IncuCyte Live-Cell Imaging

Gene-corrected fibroblast morphology and proliferation were investigated using the IncuCyte live-cell imaging device available at Imagine Institute. Briefly, primary NHFs from two different donors, RDEB-Fs treated with LV-CRISPR-N1 and LV-Donor, RDEB-Fs treated only with LV-Donor, and untreated RDEB-Fs were seeded at 10^4^ cells/well in 12-well plates and monitored during 8 days for confluence and proliferation. Cells were seeded in triplicates. Five independent regions were scanned for each well every 6 hr during 8 days with the live-cell-imaging system (phase-contrast, objective 4×). Data were analyzed using the IncuCyte S3 basic analyzer software.

### Colony-Forming Efficiency Assay

Untreated and genetically corrected RDEB-Ks (initially at 21 days post-transduction corresponding to passage 8) were analyzed for their regenerative capacity by using the colony-forming efficiency assay originally developed by Rochat and colleagues.^66^ Briefly, untreated and genetically corrected RDEB-Ks were plated for three consecutive passages (at passages 9, 10, and 11) at a density of 500 or 100 cells in 10-cm Petri dishes coated with irradiated 3T3-J2 murine embryonic fibroblast (MEF) as a feeder layer. After 13 days of culture, clones were fixed, stained with 1% rhodamine, counted under a dissecting microscope, and quantified for their growth potential.

### Absolute Quantification of Residual Cas9 Transcript Level in Genetically Corrected Cells and Grafted SEs

To evaluate the persistence of Cas9 cDNA in cells after IDLV transduction, total mRNA was extracted using the RNeasy Mini Kit (QIAGEN) from bulk transduced cells (RDEB-Ks and RDEB-Fs) and from grafted SEs. cDNA was synthesized using SuperScript III First-Stand Synthesis System (Thermo Scientific). Real-time qPCR was performed on the ABI prism 7300 Real Time Detection system (Applied Biosystems), according to the manufacturer’s instructions. The absolute quantification assay using SYBR green Dye 1 was performed in triplicate using primers specific for Cas9 (Table S2).

To generate a standard curve, a 100-fold serial dilution series of the lentiCRISPR_v2 plasmid DNA, ranging from 1 to 10^9^ copies/μL, was used. The concentration of the plasmid was measured, and the corresponding copy number was calculated by the following equation: plasmid DNA (copy) = 6.02 × 10^23^ (copy/mol) × plasmid DNA amount (g)/plasmid DNA length (bp) × 660 (g/mol/bp). The Ct values were plotted against the logarithm of their initial template copy numbers. The standard curve was generated by linear regression of the plotted points. PCR amplification efficiency (E) of the Cas9-specific primer set was calculated according to the equation E = 10^1/slope^ – 1. From the slope, a high amplification efficiency of 99% was determined for Cas9 primers.

Cas9 expression in transduced cells and in grafted SEs was evaluated in triplicates. Three independent experiments were performed.

Values corresponding to Cas9 copies/μL were converted to values of Cas9 copies/cells, taking into consideration two criteria, the initial number of cells subjected to mRNA extraction and the amount of mRNA used for the cDNA synthesis, as follows: (1) up to 3.2 × 10^6^ RDEB-Ks (200 ng/μL) and up to 1.5 × 10^6^ RDEB-Fs (150 ng/μL) were subjected to mRNA extraction, which was recovered in 20 μL; thus, the mRNA concentration corresponds to 160,000 and 75,000 cells/μL for RDEB-Ks and RDEB-Fs, respectively; (2) up to 1 μg RNA was used for cDNA synthesis, which corresponds to 5 and 6.6 μL for RDEB-Ks and RDEB-Fs, respectively; thus, 800,000 (RDEB-K) and 495,000 (RDEB-F) cells were used for cDNA synthesis; and, (3) in RDEB-K samples, 1.39 and 1.8 Cas9 copies/μL were found, which correspond to 2.25 × 10^−6^ and 1.73 × 10^−6^ Cas9 copies/cell; in RDEB-F samples, 1.89 and 1.87 Cas9 copies/μL were found, which correspond to 3.81 × 10^−6^ and 3.7 × 10^−6^ Cas9 copies/cell.

### Immunofluorescence Analysis

Gene-corrected and RDEB cells were grown for 48 hr in the presence of 20 ng/mL transforming growth factor β2 (Abcam), fixed on methanol, and stained with a monoclonal anti-C7 antibody (clone LH7.2) (Sigma-Aldrich). C7 in primary RDEB-Ks and RDEB-Fs was detected by using a fluorescein isothiocyanate (FITC)-conjugated goat-anti-mouse secondary antibody. Cells in 3 independent experiments were photographed and analyzed. For analysis of skin grafts, optimal cutting temperature compound (OCT)-embedded cryosections were fixed with acetone and stained with LH7.2 primary antibody against C7. Fluorescence-mounting medium containing DAPI (0.5 μg/mL) was used. Three different cryosections for each skin graft were analyzed by immunostaining of C7, and images were captured with a Leica TCS SP8 confocal microscope at the Cell Imaging facility of Imagine Institute. Fluorescence intensity-based quantification of C7 was performed using ImageJ software. Three independent areas for each sample were quantified for mean intensity of fluorescence. Percentage of C7 rescue was calculated by considering the mean fluorescence intensity in control skin grafts (composed of NHKs and NHFs) as 100%.

### SE Production and Grafting onto Nude Mice

SE production was performed as described previously with minor modifications.^23^, ^24^ For this study, three types of SEs were produced: (1) control SE composed of primary NHKs and NHFs, (2) RDEB SE made of patient-derived RDEB-Ks and RDEB-Fs, and (3) gene-corrected RDEB SE made of patient-derived gene-corrected RDEB-Ks and RDEB-Fs. Briefly, freeze-dried fibrinogen, obtained from human plasma of a blood donor, was reconstituted with 6 mL sterile PBS and mixed with 1.5 × 10^6^ fibroblasts in 16 mL DMEM supplemented with 10% fetal calf serum, 42 μg/mL tranexamic acid, and 10 μg/mL thrombin (Sigma-Aldrich). The fibrin-fibroblast mix was dispensed into a 6-well culture plate, and a day later 1.7 × 10^6^ keratinocytes in 18 mL DMEM supplemented with 50 μg/mL ascorbic acid were seeded on top (3 mL per well). Then 15 days later, SEs were grafted onto the dorsal region of immunodeficient NMRI-Foxn1nu/Foxn1nu nude mice (n = 18) using a skin flap procedure, as previously reported.^23^, ^24^ 1 and 2 months later, mice were sacrificed and skin grafts were taken for skin immunohistochemistry and molecular analysis. To evaluate gene correction efficiency in grafted SE, 15 cryosections of 5-μm thickness were subjected to genomic DNA extraction using Genomic DNA from tissue kit (Macherey-Nagel). 50 ng genomic DNA of corresponding samples was subjected to ddPCR using primers and probes complementary to the corrected or the mutated sequence in exon 2 of *COL7A1* (custom design), as already described above.

The animal experiments were performed in compliance with guidelines for animal experiments in France and conducted in accordance with the ethical principles.

### Off-Target Site Analysis

Potential off-target sequences for gRNA N1 were identified by ZiFiT Targeter 4.2 (http://zifit.partners.org) in the human genome. Only 3 potential off-targets (Off-Targets_1,2,3) were found with 2 MMs in non-coding regions by ZiFiT Targeter 4.2. We also predicted off-targets on http://crispr.mit.edu, and we identified 3 potential off-targets with 3 MMs (Off-Targets_4,5,6) and 2 potential off-targets with 4 MMs (Off-Targets_7,8) in coding regions. Overall, 8 off-target sequences were subjected to the Surveyor cleavage assay. The genomic DNA from RDEB-Ks treated with IDLVs encoding for the LV-CRISPR-N1 (2 pg p24 per cell) and from genetically corrected RDEB-Ks treated with IDLVs encoding for the LV-CRISPR-N1 and the LV-Donor (2 pg p24/cell and 0.5 pg p24/cell) at 21 days post-transduction was extracted, and the corresponding regions were amplified by PCR using specific primers (Table S4). The Surveyor cleavage assay was performed following the manufacturer’s instructions (Integrated DNA Technologies) at each potential off-target hit.

### Transmission Electron Microscopy

For ultrastructural analysis, the central piece of each skin graft (approximately 2 mm^3^) was dissected and immediately fixed with Karnovsky’s fixative (2% formaldehyde and 4% glutaraldehyde in cacodylate buffer). The ultrastructural analysis was performed at the electron microscopy core facility in Cochin Institute, Paris.

### Ethics Statement

Informed consent was obtained from the RDEB patient homozygous for the c.189delG; (p.Leu64Trpfs*40) null mutation in exon 2.

## Author Contributions

A.I. designed, performed experiments, and interpreted data. C.G. was involved in 3D skin equivalent production and grafting experiments. M.B. was involved in CRISPR/Cas9-related gRNA design and cloning into MLM3636 plasmid. A.S. was involved in ultrastructural analysis of grafted skin samples. F.M. gave scientific advice. A.H. supervised the project. A.I. and A.H. wrote the paper.

## Supplemental Information

Document S1. Figures S1–S6 and Tables S1–S4

Document S2. Article plus Supplemental Information
