# Supplementary material for: Ex Vivo COL7A1 Correction for Recessive Dystrophic Epidermolysis Bullosa Using CRISPR/Cas9 and Homology-Directed Repair
Source: Mol Ther Nucleic Acids. 2018 Jun 26;12:554–67. doi: 10.1016/j.omtn.2018.06.008 (PMC6077132; doi:10.1016/j.omtn.2018.06.008)
Supplement: Document S1. Figures S1–S6 and Tables S1–S4 [file mmc1.pdf]

OMTN, Volume *12*

Supplemental Information

Ex Vivo COL7A1 Correction for Recessive Dystrophic Epidermolysis Bullosa Using CRISPR/Cas9 and Homology-Directed Repair

Araksya Izmiryan, Clarisse Ganier, Matteo Bovolenta, Alain Schmitt, Fulvio Mavilio, and Alain Hovnanian

Figure S1

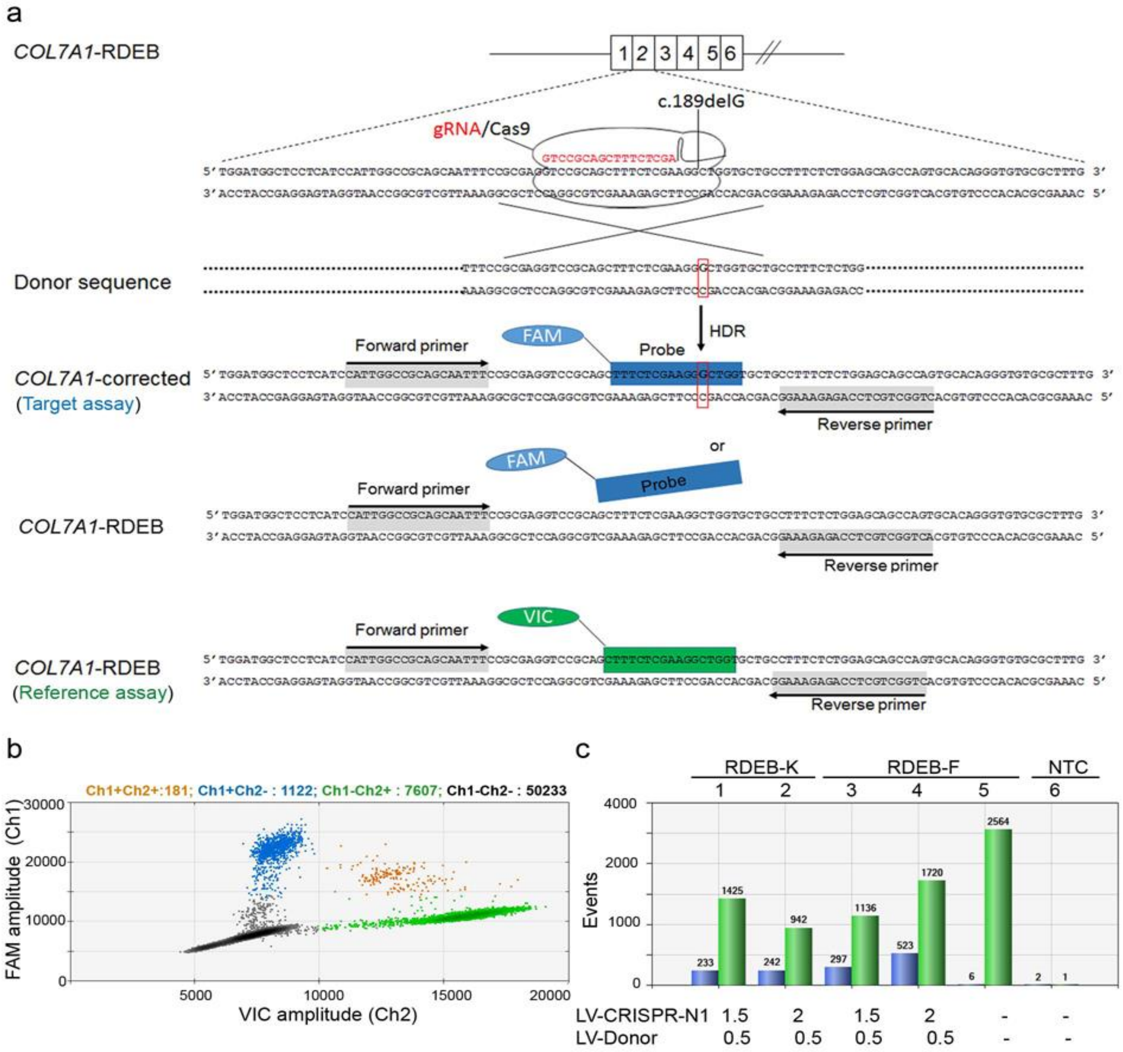

Detection of CRISPR/Cas9-mediated HDR at the *COL7A1* locus by Taqman-ddPCR on the genomic DNA level. (a) Gene editing detection strategy for *COL7A1* by ddPCR. Location of a common primer pair and allele-specific probes conjugated with FAM (specific for the corrected *COL7A1*) or VIC (specific for the mutated *COL7A1*) fluorophores are indicated. (b) 2-D fluorescence amplitude plot generated by Quantasoft software showing walls containing both of corrected and mutant *COL7A1*. The black cluster on the plot represents the negative droplets (Ch1-Ch2-), the blue cluster represent the droplets that are positive for the corrected *COL7A1* only (Ch1+Ch2-), the green cluster represents the droplets that are positive for the mutant *COL7A1* only (Ch1-Ch2+) and the orange cluster represents the droplets that are positive for both (Ch1+Ch2+). (c) The 'Events' histogram shows the total number of droplets positive for FAM (in blue) and VIC (in green) signals which correspond to the corrected or mutated *COL7A1*, respectively. Ch1: channel 1, corresponds to the FAM amplitude; Ch2: channel 2, corresponds to the VIC amplitude.

Figure S2

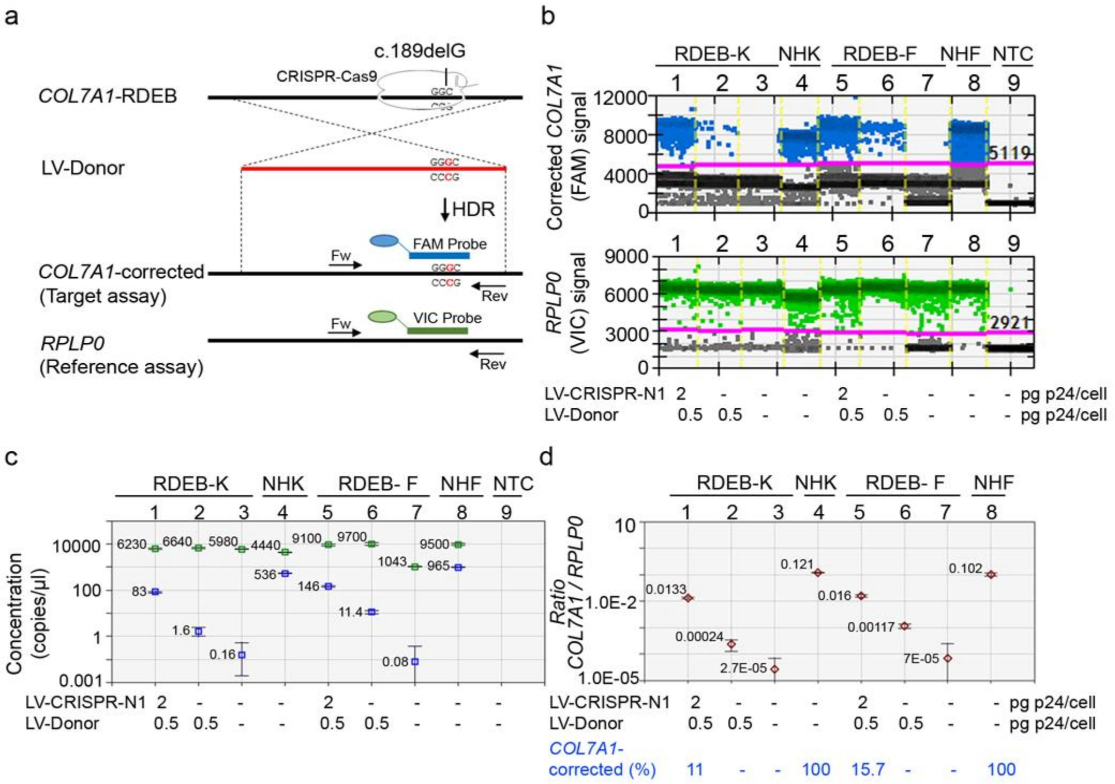

TaqMan-ddPCR-based detection of corrected *COL7A1* mRNA expression after CRISPR/Cas9-mediated HDR. (a) Gene editing detection strategy for *COL7A1* by ddPCR at the mRNA level. In the scheme, the same experimental settings as for the Figure 3a, are shown. The VIC-conjugated specific probe recognizes the housekeeping gene *RPLP0*. (b) Primary RDEB-K and RDEB-F were transduced with indicated doses of IDLVs (pg p24 per cell). 21-days post transduction, mRNA was extracted, subjected for Reverse Transcription and analyzed by Taqman-ddPCR to detect the expression of corrected *COL7A1* relative to the expression level of *COL7A1* in normal cells. 1-D fluorescence amplitude plots are shown. Yellow lines indicate borders between different samples. Blue dots correspond to the FAM signal and represent droplets containing the corrected *COL7A1*. Green dots correspond to the VIC signal and represent housekeeping *RPLP0*. Grey dots correspond to empty droplets. (c-d) Quantification of positive droplets using Quantasoft. The concentration plot, showing gene-edited wells of FAM and VIC amplicons is automatically determined by the software using the total number of events (displayed in Figure S2) by correcting for Poisson distribution. The blue markers indicate corrected *COL7A1* copies/µl and the green markers indicate housekeeping *RPLP0* copies/µl. The 'Ratio' plot shows the percentage of the corrected *COL7A1* normalized to the housekeeping *RPLP0* background (orange markers). All error bars were generated by QuantaSoft and represent a 95% confidence interval. The percentage of corrected *COL7A1* mRNA in RDEB-K and RDEB-F was calculated by considering the Ratio of *COL7A1/RPLP0* in normal cells (NHK and NHF, respectively) as 100%.

Figure S3

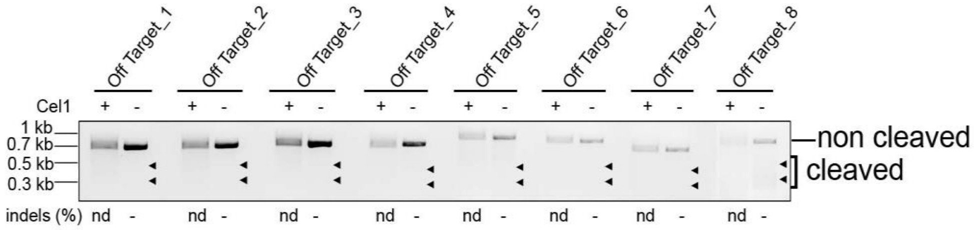

Off-target site analysis in genetically corrected RDEB-K 21 days post-transduction. Genomic DNA from corrected RDEB-K co-treated with IDLVs encoding for the LV-CRISPR-N1 (2 pg p24 per cell) and the LV-Donor (0.5 pg p24 per cell) was extracted and regions corresponding to off-target sequences were amplified by PCR using specific primers (listed in Table S4). The Surveyor cleavage assay was performed at each potential off-target hit. No Surveyor activity indicative of cleavage at predicted off-target sites was detected. nd: non detected.

Figure S4

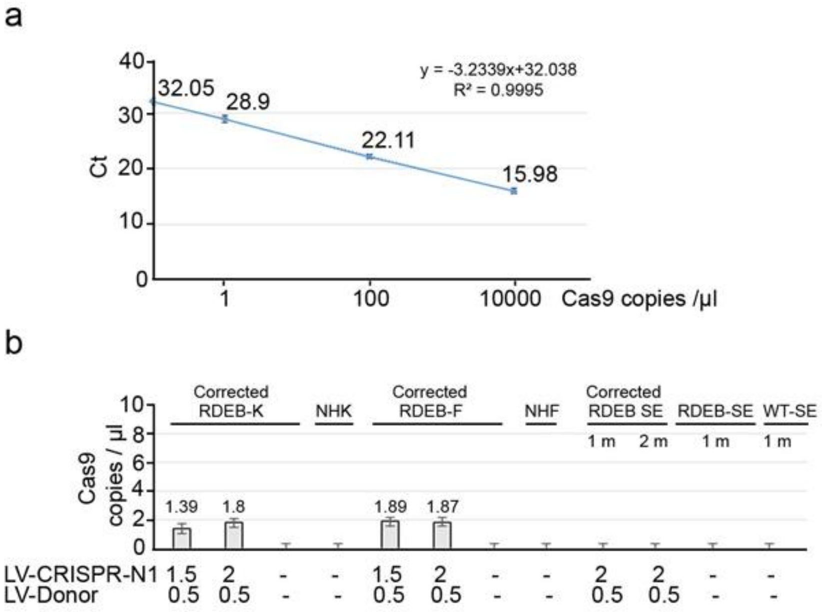

Absolute quantification the residual Cas9 transcripts in genetically corrected cells and grafted skin equivalents. (a) Standard curve of the lentiCRISPR_v2 plasmid DNA, ranging from 1 to 10^9 copies/µl. The Ct values were plotted against the logarithm of their initial template copy numbers. The standard curve was generated by linear regression of the plotted points. (b) To evaluate the persistence of Cas9 cDNA in cells after IDLV transduction, total mRNA was extracted and cDNA was synthetized from bulk transduced RDEB-K, RDEB-F and from grafted skin equivalents. Cas9 expression in transduced cells and in grafted skin equivalents was evaluated in triplicates. Three independent experiments were performed.

Figure S5

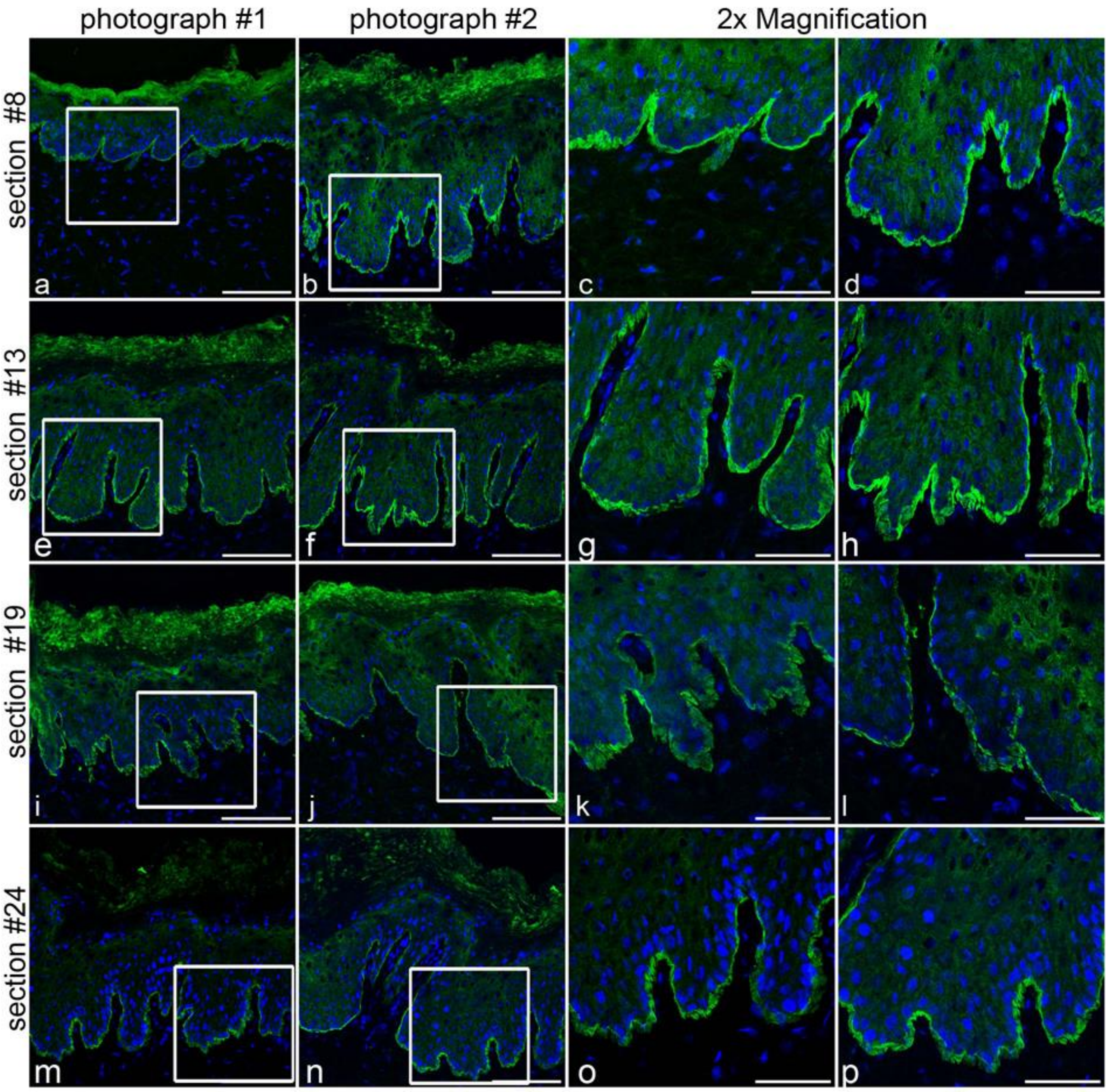

Type VII collagen rescue, localization and AF formation at the dermal-epidermal junction in serial sections of the genetically corrected skin grafts. Immunofluorescence analysis of grafted SE composed of genetically corrected primary RDEB keratinocytes and fibroblasts at 2 months after deflaping. Skin samples composed of genetically corrected cells showed re-expression and normal localization of C7 at the dermal-epidermal junction in serial sections of SE. Scale bar = 100 µm.

Figure S6

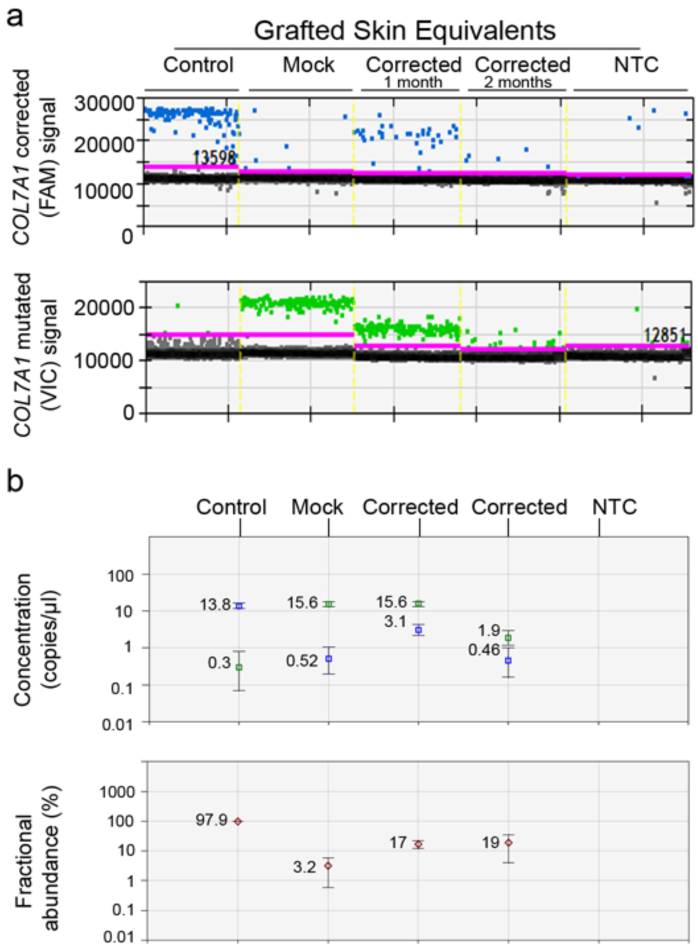

Detection of CRISPR/Cas9-mediated HDR at the *COL7A1* locus by Taqman-ddPCR on the genomic DNA level in skin grafts. See Figure S1 for the experimental settings. (a) 1 month or 2 months post-grafting, gDNA was extracted from cryosections and analyzed by ddPCR to assess allelic frequency of corrected *COL7A1* on the mutated background in skin grafts. 1-D fluorescence amplitude plots are shown for FAM and VIC signals. Blue dots correspond to the FAM signal amplitude and represent droplets containing the normal or corrected *COL7A1* alleles. Green dots correspond to the VIC signal amplitude and represent the mutated *COL7A1* alleles. Grey dots correspond to empty droplets. Yellow lines indicate borders between different samples. (b) Quantification of positive droplets using Quantasoft Software. The concentration plot, showing gene-edited wells of FAM and VIC amplicons is automatically determined by the software using the total number of events by correcting for Poisson distribution. The blue markers indicate corrected *COL7A1* copies/µl and the green markers indicate mutated *COL7A1* copies/µl. The Fractional abundance plot shows the percentage frequency of the corrected *COL7A1* on the mutated *COL7A1* background. All error bars were generated by QuantaSoft and represent a 95% confidence interval.

Table S1. Sequences of guide RNAs

gRNA	Sequence	gRNA length (bp)	Strand	Cut-to-mutation distance* (bp)	Off target activity	Surveyor digestion product (bp)
N1	GTCCGCAGCTTTCTCGA	17	First Strand	5 (downstream)	0 MMs = 1<br>1 MMs = 0<br>2 MMs = 3	684 (non cleaved)<br>379 (cleaved)<br>305 (cleaved)
N2	GAAAGCTGCGGACCTCG	17	Reverse Strand	21 (downstream)	0 MMs = 1<br>1 MMs = 0<br>2 MMs = 6	684 (non cleaved)<br>391 (cleaved)<br>293 (cleaved)
N3	GATGGCTCCTCATCCAT	17	First Strand	43 (downstream)	0 MMs = 1<br>1 MMs = 0<br>2 MMs = 18	684 (non cleaved)<br>340 (cleaved)<br>344 (cleaved)
N4	GCGCACACCCTGTGCAC	17	Reverse Strand	31 (upstream)	0 MMs = 1<br>1 MMs = 0<br>2 MMs = 14	684 (non cleaved)<br>444 (cleaved)<br>240 (cleaved)
N5	GCTGCGGCCAATGGATG	17	Reverse Strand	44 (downstream)	0 MMs = 1<br>1 MMs = 1<br>2 MMs = 14	684 (non cleaved)<br>366 (cleaved)<br>318 (cleaved)

Table S2. Absolute quantification of residual Cas9 cDNA expression in genetically corrected RDEB keratinocytes, fibroblasts and in grafted skin equivalents (SE)

		Experience N1	Experience N2	Experience N3	Mean Experiences N1-N2-N3	
Sample Name	Target gene	Mean Ct	Mean Ct	Mean Ct	Mean Ct	Cas9 copies/µl
RDEB-K (IDLVs : 1.5 / 0.5)	Cas9	27.4	27.76	27.42	27.52	1.39
RDEB-K (IDLVs : 2 / 0.5)	Cas9	26.07	26.97	25.54	26.19	1.8
RDEB-K	Cas9	32.52	32.98	32.01	32.5	-
NHK	Cas9	35.9	34.05	34.04	34.66	-
RDEB-F (IDLVs : 1.5 / 0.5)	Cas9	25.63	26.22	25.91	25.92	1.89
RDEB-F (IDLVs : 2 / 0.5)	Cas9	26.07	26.09	25.73	25.96	1.87
RDEB-F	Cas9	Undetermined	Undetermined	Undetermined	Undetermined	-
NHF	Cas9	Undetermined	Undetermined	Undetermined	Undetermined	-
Corrected-SE-1 month - N1	Cas9	32.33	Undetermined	Undetermined	32.33	-
Corrected-SE-1 month - N2	Cas9	Undetermined	Undetermined	Undetermined	Undetermined	-
Corrected-SE-1 month - N3	Cas9	Undetermined	Undetermined	Undetermined	Undetermined	-
Corrected-SE-2 months - N1	Cas9	39.34	Undetermined	Undetermined	39.34	-
Corrected-SE-2 months - N2	Cas9	36.94	33.18	Undetermined	35.06	-
Corrected-SE-2 months - N3	Cas9	Undetermined	Undetermined	31.4	31.4	-
RDEB-SE-1 month - N1	Cas9	Undetermined	37.27	Undetermined	37.27	-
RDEB-SE-1 month - N2	Cas9	Undetermined	30.88	34.83	32.85	
WT-SE-1 month - N1	Cas9	39.44	Undetermined	Undetermined	39.44	-
WT-SE-1 month - N2	Cas9	Undetermined	Undetermined	Undetermined	Undetermined	-

Table S3. HDR efficiency in genetically corrected RDEB cells and grafted skin equivalents (SE)

Cells/Grafts	Genetically corrected RDEB-K			Genetically corrected RDEB-F			Grafted SE	
Sample	gDNA	cDNA	Protein	gDNA	cDNA	Protein	gDNA	Protein
Assay	ddPCR	ddPCR	WB	ddPCR	ddPCR	WB	ddPCR	IF
Figure	N3	S2	N5	N3	S2	N5	N6, S6	N6, S6
Correction	19.6%	11%	11%	22.1%	15.7%	-	17-19%	20-26%

Table S4. Oligonucleotides and Probes sequences

Figure	Assay	Primer name	Sequence
N1	NHEJ actitvity	Fw_Surveyor	GTCCCCTGCCTTATGCCAA
N1	NHEJ actitvity	Rev_Surveyor	GCACCTTCCTGTCTTGCAGT
N2	Allele specific PCR	Commun_P1	GATTCCTCCTAATTCTGGGACTC
N2	Allele specific PCR	Mutant_P2	GCACCTTCCTGTCTTGCAGTAG
N2	Allele specific PCR	Corrected_P3	GCACCTTCCTGTCAGCAAGTGA
N2	Allele specific PCR	Fw_GAPDH	TCCATGCCAT CACTGCCACCCAG
N2	Allele specific PCR	Rev_GAPDH	CATACCAGGAAATGAGCTTGACAAAGT
N3; S1, 2, 5	TaqMan-ddPCR	Fw_Exon2	CATTGGCCGCAGCAATTT
N3; S1, 2, 5	TaqMan-ddPCR	Rev_Exon2	CTGGCTGCTCCAGAGAAAGG
N3; S1, 2, 5	TaqMan-ddPCR	FAM_Probe	TTTCTCGAAGGGCTG-MGB
N3; S1, 5	TaqMan-ddPCR	VIC_Probe	CTTTCTCGAAGGCTGGT-MGB
N6, S3	Off-target activity	Fw_OT1	GCTGCCTTCTCGTACTCACA
N6, S3	Off-target activity	Rev_OT1	TGCCTTTCATAGGGAGTGCTG
N6, S3	Off-target activity	Fw_OT2	AATTCTGCTTGTGGCTGCAC
N6, S3	Off-target activity	Rev_OT2	ACCACGATTGGACTAGAAGGC
N6, S3	Off-target activity	Fw_OT3	AGGTTCACGAGGCTGTAACG
N6, S3	Off-target activity	Rev_OT3	TCTGCTAGACACCCCTCCTC
N6, S3	Off-target activity	Fw_OT4	GTCGCTTTGCTTGCTCTCTG
N6, S3	Off-target activity	Rev_OT4	ACTTCAGCAACTGGAGAGGC
N6, S3	Off-target activity	Fw_OT5	GATAAGAAATGAGGTAATGC
N6, S3	Off-target activity	Rev_OT5	CACAGCAAGAATACATCATCTA
N6, S3	Off-target activity	Fw_OT6	CCAGGGCAAGGGTCTTTCTC
N6, S3	Off-target activity	Rev_OT6	TTTGCTGGGCTACTTTGCAG
N6, S3	Off-target activity	Fw_OT7	TCCCAAGTTAGGAGGGGTCA
N6, S3	Off-target activity	Rev_OT7	CCAGAAATGGAGTGGGCTGT
N6, S3	Off-target activity	Fw_OT8	GGGACACATGTGCAGACTCA
N6, S3	Off-target activity	Rev_OT8	GAGCCATCTGCAGGGTTTGT
S4	Absolute qPCR	Fw_Cas9	GGACTCCCGGATGAACACTAAG
S4	Absolute qPCR	Rev_Cas9	AAAGTGCGCGAGATCAACAAC
